# Ankle injury rehabilitation (AIR): a feasibility randomised controlled trial comparing functional bracing to plaster cast in the treatment of adult ankle fractures

**DOI:** 10.1186/s40814-019-0441-6

**Published:** 2019-04-17

**Authors:** Rebecca S. Kearney, Rebecca McKeown, Daniel Gallacher, Jaclyn Brown, Dipesh Mistry, Nick Parsons, Jonathan Young, Matthew Costa

**Affiliations:** 1 Clinical Trials Unit, University of Warwick, Gibbet Hill Road, Coventry, CV4 7AL England; 2 Warwick Medical School, University of Warwick, Gibbet Hill Road, Coventry, CV4 7AL England; 3 University Hospitals Coventry and Warwickshire, Clifford Bridge Rd, Coventry, CV2 2DX England; 4 Trauma Unit, Kadoorie Centre, John Radcliffe Hospital, Level 3, Oxford, OX3 9DU England

**Keywords:** Ankle, Fracture, Rehabilitation, Immobilisation

## Abstract

**Background:**

Approximately 9% of a trauma surgeon’s workload in the UK is managing ankle fractures. Following an ankle fracture immobilisation with a plaster cast or removable orthotic is usual. The aim of this research was to assess the feasibility of a large multi-centre randomised controlled trial (RCT) to evaluate the difference between plaster cast and a removable orthotic for the management of adults with an ankle fracture.

**Methods:**

A feasibility randomised controlled trial was undertaken in a UK trauma hospital in adults with an ankle fracture for which the treating clinician would consider plaster cast a reasonable management option. Exclusions included open or pathological fracture, unable to adhere to trial procedures, had other lower limb injury or required close contact casting. Participants were randomised using an independent telephone service to receive either plaster cast or removable orthotic. The primary outcome was to determine the recruitment and follow-up rates at 6 weeks, 3 and 6 months to assess the feasibility of a full RCT.

**Results:**

Eighty five eligible patients presented during the 10-month recruitment period, 50 consented. Two patients were randomised who did not fulfil the eligibility criteria (protocol deviations), and 1 patient from each group crossed over. Follow-up at each time point was 92% at 6 weeks; 74% at 3 months and 83% at 6 months.

**Conclusions:**

Recruitment and follow-up data demonstrated feasibility of conducting a larger-scale randomised controlled trial. The distributional properties of the patient-reported outcome measures will be used to determine future sample sizes.

**Trial registrations:**

This study is registered with the ISRCTN (ISRCTN17809322), assigned 5 November 2015 and approved by the NRES Committee (The Black Country, 15/WM/0340), protocol version 2.0 (17 November 2015). It is co-sponsored by the University Hospitals Coventry and Warwickshire NHS Trust and University of Warwick and funded by the NIHR Research for Patient Benefit (PB-PG-0614-34,009). The trial sponsors have no direct involvement in any aspects of study design, conduct or decision to submit the report for publication.

## Background

Approximately 9% of a trauma surgeon’s workload in the UK is managing ankle fractures, occurring in 122 per 100,000 persons [1]. This injury causes severe loss of function, most notably in the elderly population of patients [2]. It is estimated that over half of patients still experience symptoms up to 3 years post-injury, with this rate being higher in patients over 40 years of age [3]. The incidence of ankle fractures is set to rise along with the ageing population which will place significant burden on healthcare systems [4].

Traditionally, the immediate rehabilitation has been plaster cast immobilisation for several weeks. A cast provides maximum support; however, there are risks associated with prolonged immobilisation such as muscle atrophy, deep vein thrombosis and joint stiffness. There are also long-term consequences, including gait abnormalities, persistent calf muscle weakness and ultimately a permanent deficit in pre-injury patient-reported functional outcome [5]. Alternative fixed angle removable orthotic may potentially address these issues [6, 7]. The orthotic immobilises the fracture but can be removed to allow for regular mobilisation of the foot and ankle, which may lessen some of the risks involved with prolonged immobilisation in plaster cast. However, it is unknown whether further complications, such as loss of fracture reduction and alignment may be increased with functional bracing.

In 2010, an orthopaedic trauma network (AOUK) undertook a research priority exercise [8]. One of the top priority questions was to establish if there is a clinical advantage to wound healing and ankle function of different rehabilitation plans following an ankle fracture. This was followed by a 2012 Cochrane review [9] highlighting the need for further research into the optimal method of immobilisation for both operatively and non-operatively managed acute ankle fractures. Finally, this issue was raised again in a James Lind Alliance Priority Setting Partnership in 2018, which included ‘What is the best physiotherapy regime for adults during out-of-hospital recovery from a fragility fracture of the lower limb?’ in its top 10 priority research questions [10].

Delivering full-scale multi-centre randomised controlled trials (RCT) in trauma and orthopaedics cost large amounts of funding (typically in excess of £1 M) and large amounts of time (typically 4–5 years) [11, 12] a preliminary feasibility study provides essential information to evaluate if a full RCT is achievable and refine trial procedures ahead of the full RCT. The objectives of this feasibility RCT were as follows:

1. Evaluate the distributional properties of the Manchester-Oxford Foot and Ankle Questionnaire (MOXFQ) in order to estimate the likely sample size required for a full randomised controlled trial.
2. Evaluate the number of eligible patients within the recruiting site.
3. Evaluate the willingness of clinicians to recruit participants (the proportion of eligible patients who are offered participation in the study).
4. Evaluate the willingness of patients to be randomised (the proportion of eligible patients who agree to participate in the study).
5. Evaluate the follow-up and response rates to questionnaires.
6. Refine the statistical analysis plan to provide the most efficient and sensitive analysis.
7. To discuss the feasibility trial at a national consensus meeting to inform the design of a full RCT.

## Methods

This is a single-centre feasibility randomised controlled trial. A full study protocol has been previously published by ‘*Pilot and Feasibility Studies’* [13]. The recruitment site opened 10 November 2015 with criteria that included all adults presenting to a single UK Major Trauma Centre with an ankle fracture managed operatively with open reduction and internal fixation. The Trial Management Group (TMG) changed the entry criteria on 13 January 2016 to also include participants who did not require surgical fixation, with entry criteria being that the treating clinician felt that plaster cast would be a reasonable management option. This decision was made after the TMG gained a clinical consensus at the recruiting site that clinicians would be willing to randomise both operatively and non-operatively managed patients.

Exclusion criteria were patients who were unable to walk prior to injury, those with a previous ankle fracture randomised in the current trial, those with open ankle fractures, pathological ankle fractures (e.g. from known metastatic disease), patients who would be unable to adhere to trial procedures or complete questionnaires, any other lower limb injury (including bilateral ankle fractures and syndesmosis injury requiring surgery) and patients who, in the opinion of the treating clinician, would require close contact casting.

Screening logs were completed to assess the number of eligible potential participants, main reasons for exclusion, the number of eligible and participant unwilling and the number of eligible and clinician unwilling. Patients who were deemed eligible were invited to take part by a member of the research team, who provided the patient with an information sheet. Written informed consent was then obtained if the patient agreed to take part. Clinicians reviewed the baseline X-ray and classified the fractures according to the Weber Ankle Fracture Classification [14], recorded as part of the baseline data set.

Participants who consented were randomised to either a plaster cast or fixed angle removable orthotic on a 1:1 basis using a secure computer generated randomisation sequence, administered via an independent telephone service. Randomisation was stratified by age (above and below 50 years), and whether or not the patient had surgery. These were to account for changes in the bone density in the over 50 population [15] and injury severity respectively. Due to the type of intervention, it was not possible to blind the participants to their allocation. A research nurse at the site was responsible for enrolling and assigning participants to the randomised intervention.

All participants who required an open reduction and internal fixation of their fracture received this according to the preferred operative technique of the treating surgeon. Patients were then immobilised in a back slab until removal of the stitches, approximately 10 days post-surgery, at which point randomisation occurred. The weight-bearing status was left to the discretion of the treating clinician. All non-operative patients were approached to enter the trial on first presentation to the trauma clinic.

Participants in the control group were immobilised in a plaster cast or fibre glass cast. Participants in the interventional group received immobilisation in a fixed angle removable orthotic, typically a plastic boot with a padded inner part, which is held in place with Velcro straps. The brand and manufacturer of the brace was not specified. Participants in this group were encouraged to remove their orthotic regularly and were given an exercise sheet detailing two simple unloaded range of movement exercises. They were advised to do 10 repetitions of each, three times per day. The participants in this group were given a diary to record compliance with exercises.

Following the period of immobilisation (typically 6 weeks), all participants received the same standardised written physiotherapy advice. The decision to formally refer the patients to outpatient physiotherapy was left to the discretion of the treating clinician.

A formal sample size calculation (power analysis) was not considered appropriate for this feasibility study. However, data relating to the distributional properties of the planned patient-reported outcome measures to be used in a full RCT were evaluated to inform future sample size calculations.

Participants were reviewed at 6 weeks and 6 months in clinic and via postal questionnaire at 3 months. The patient-reported outcome measures collected were

MOXFQ [16]: a validated questionnaire which is self-reported. It contains 16 items, each with 5 response options comprising 3 separate underlying dimensions: walking/standing problems (7 items), foot pain (5 items) and issues related to social interaction (4 items). Item responses are each scored from 0 to 4, with 4 representing the most severe state. The scale scores representing each dimension are produced by summing the responses to each item within that dimension. Raw scale scores are then converted to a metric (0–100; 100 = most severe).

Olerud and Molander Ankle Score (OMAS) [17]: is a self-administered questionnaire. The score is based on nine different items: pain, stiffness, swelling, stair climbing, running, jumping, squatting, supports and work/activities of daily living. The scale from 0 point (totally impaired function) to 100 points (completely unimpaired function).

EuroQol EQ-5D-5 L [18]: consists of 5 dimensions each with a 5-level answer possibility. Each combination of answers is converted into a health utility score.

Complications and plain radiographs at baseline, 6 weeks and 6 months were also collected. Baseline outcome scores for pre- and post-injury status were collected in clinic along with baseline demographic data.

Participant characteristics and outcomes were summarised as mean and standard deviation (SD) for continuous data and frequency and percentage (%) for categorical data. No between group comparison tests were pre-planned, as this study it is not designed to infer significance to any observed treatment differences. Instead, the distributions of the patient-reported outcome measures and participant recruitment and retention rates were examined to inform the design of a full RCT, as per the outlined objectives of this study.

## Results

**Fig. 1:**
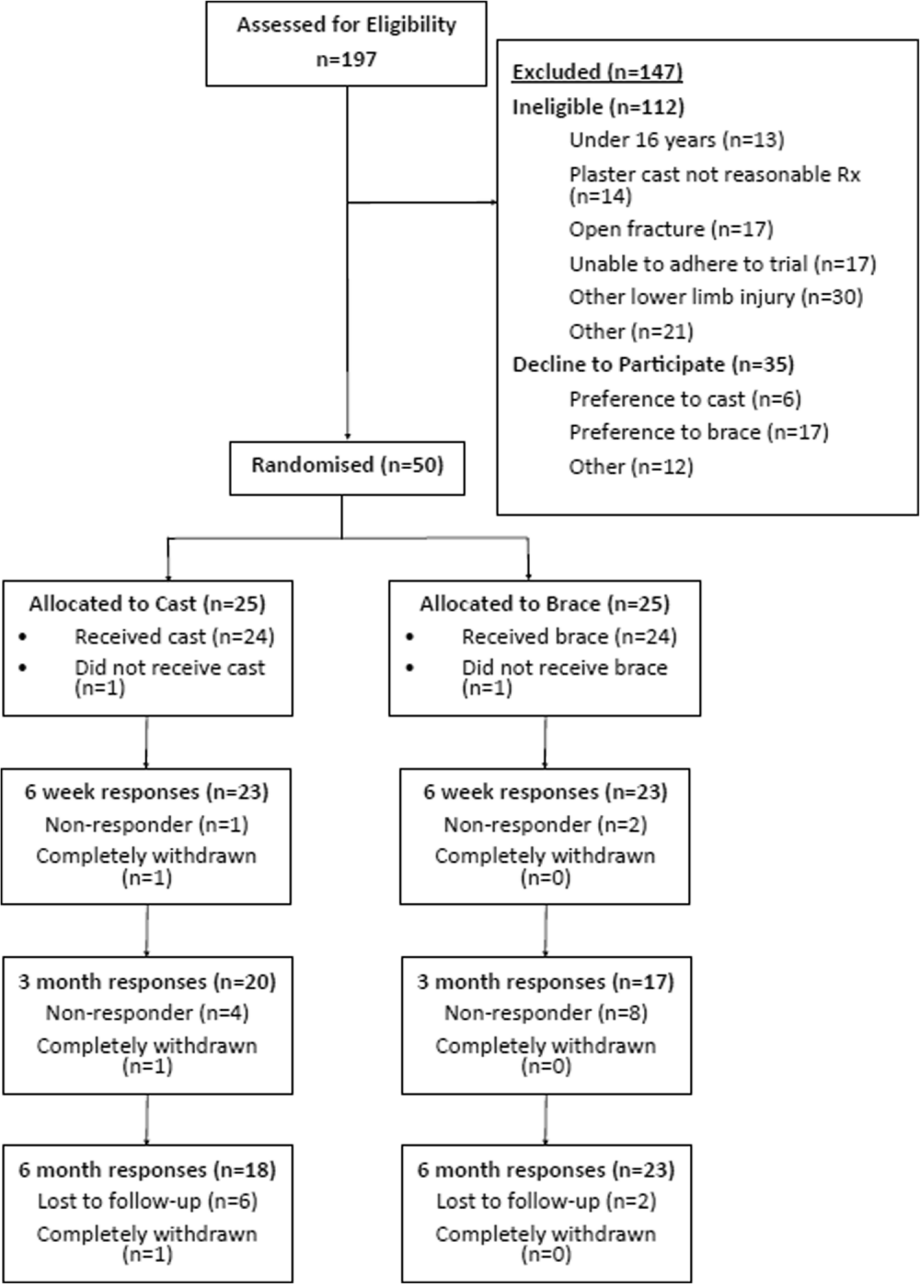
CONSORT flow diagram

Table 1 shows the baseline demographics of the trial participants and Fig. 1 shows the flow of participants in a CONSORT diagram. All data in tables are presented as ‘Arm A’ and ‘Arm B’ in order to avoid making inferences concerning differences between trial arms. Trial arms were unblinded in the CONSORT diagram in order to further understand cross over, withdrawal and loss to follow-up.

**Table 1:** Participant baseline data at recruitment; means (standard deviation) are shown for continuous outcomes and counts and percentages for categorical outcomes

Characteristic	Arm A (*n* = 25)	Arm B (*n* = 25)	Total (*n* = 50)
Age (years)	42.7 (14.9)	40.7 (17.1)	41.7 (15.9)
Gender male, *n* (%)	14 (56%)	10 (40%)	24 (48%)
Gender female, *n* (%)	11 (44%)	15 (60%)	26 (52%)
Height (cm)	170.8 (10.0)	171.3 (10.7)	171.1 (10.2)
Weight (kg)	87.9 (24.0)	84.2 (18.4)	86.2 (21.4)
Fracture of the lateral malleolus, *n* (%)	21 (84%)	21 (84%)	42 (84%)
Fracture of the medial malleolus, *n* (%)	8 (32%)	10 (40%)	18 (36%)
Fracture of the posterior malleolus, *n* (%)	3 (12%)	3 (12%)	6 (12%)
Weber classification, *n* (%)			
A	1 (4%)	1 (4%)	2 (4%)
B	18 (72%)	17 (68%)	35 (70%)
C	2 (8%)	3 (12%)	5 (10%)
Operative patient, *n* (%)	15 (60%)	16 (64%)	31 (62%)
Previous problems with the lower limb on the injured side, *n* (%)	3 (12%)	8 (32%)	11 (22%)
Side of Injury, *n* (%)			
Right	14 (56%)	15 (60%)	29 (58%)
Left	11 (44%)	10 (40%)	21 (42%)
Patients diagnosed with diabetes, *n* (%)	2 (8%)	1 (4%)	3 (6%)
Patient regular and current smoker	6 (24%)	7 (28%)	13 (26%)

**Fig. 2:**
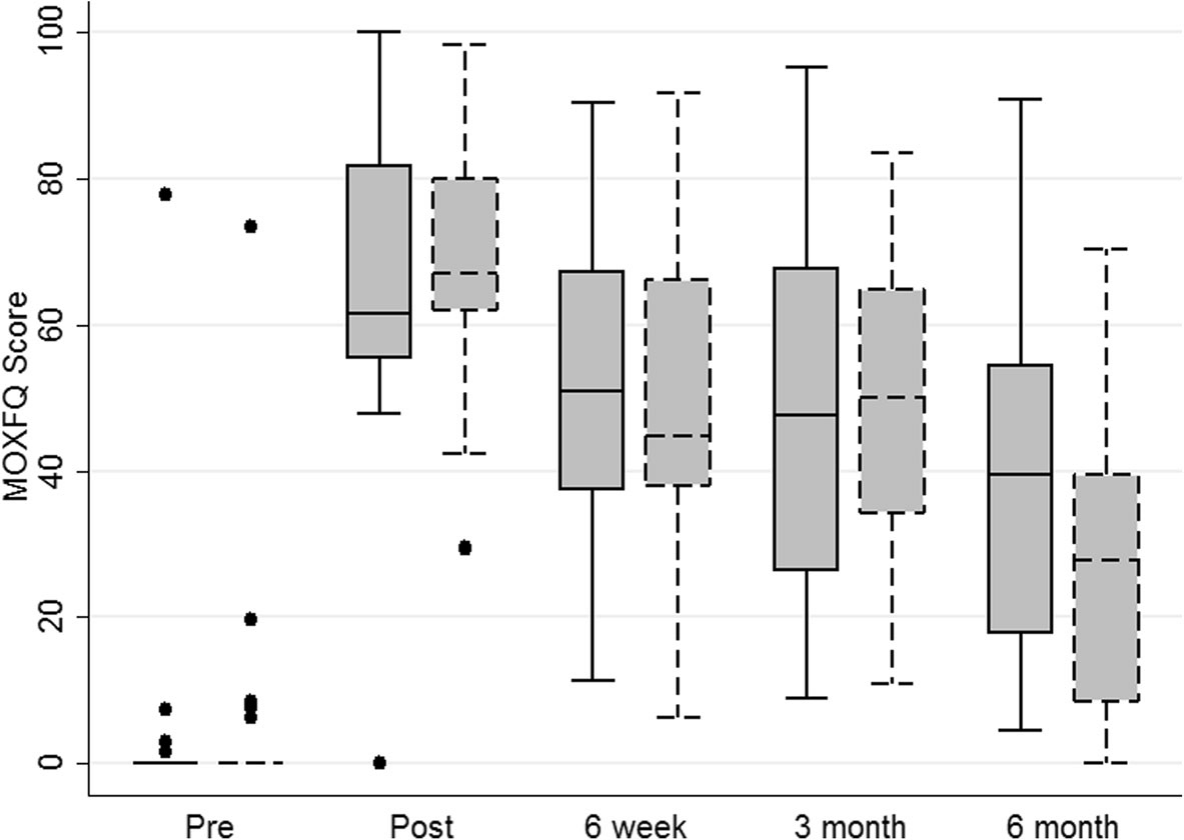
MOXFQ box plots

**Fig. 3:**
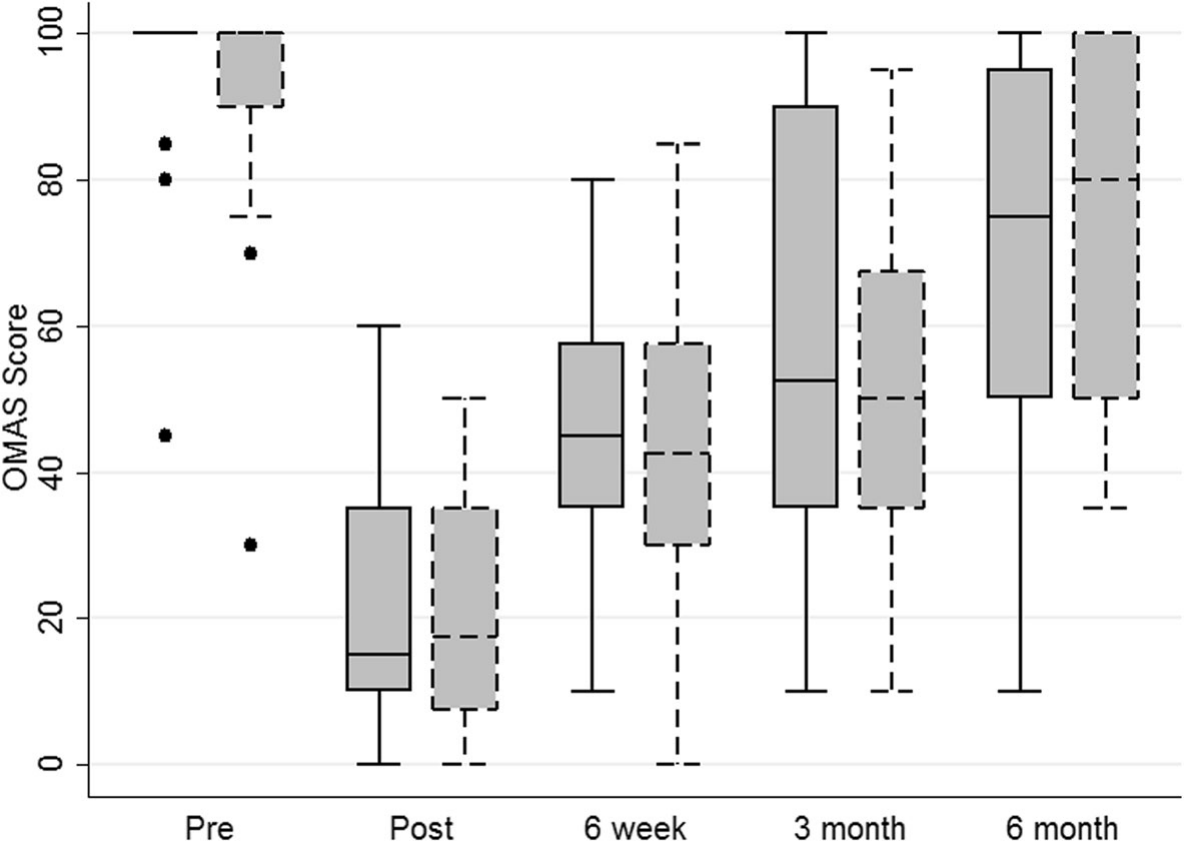
OMAS box plots

**Fig. 4:**
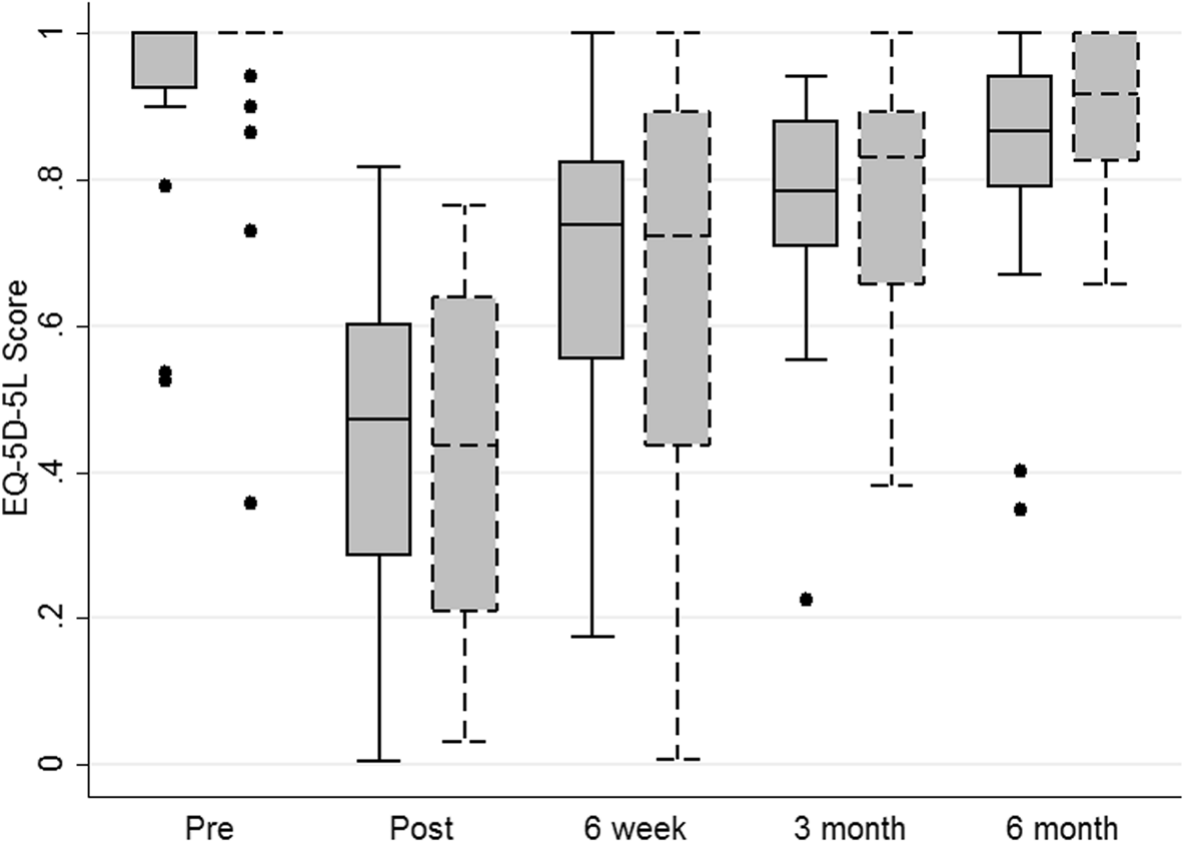
EQ-5D-5 L box plots

Objective 1 was to evaluate the distributional properties of the MOXFQ in order to estimate the likely sample size required for a full RCT. Table 2 and Figs. 2, 3 and 4 illustrate the distributional properties of the MOXFQ, OMAS and EQ-5D-5 L outcome measures collected at baseline and at each follow-up time point. The standard deviations (SD) of the pooled 6-month follow-up data were 25.0 and 26.1 for MOXFQ and OMAS respectively.

**Table 2:** Summary statistics of outcome data; entries are presented as mean (standard deviation)

	Arm A	Arm B	Total
Pre-injury scores			
MOXFQ	3.7 (15.5	4.6 (15.	4.2 (15.1) *n* = 50
OMAS	95.4 (12.4)	91.7 (16.	93.5 (14.3) *n* = 50
EQ5D5L	0.93 (0.13)	0.95 (0.14)	0.44 (0.23) *n* = 50
Post injury scores			
MOXFQ	66.5 (20.6)	67.9 (16.8)	67.2 (18.6) *n* = 50
OMAS	21.0 (17.7)	21.0 (16.2)	21.0 (16.9) *n* = 50
EQ5D5L	0.44 (0.23)	0.45 (0.23)	0.44 (0.23) *n* = 50
6 weeks			
MOXFQ	51.5 (18.7)	50.1 (21.3)	50.8 (19.9) *n* = 46
OMAS	45.0 (18.1)	44.5 (21.1)	44.8 (19.4) *n* = 46
EQ5D5L	0.68 (0.22)	0.67 (0.28)	0.68 (0.25) *n* = 46
3 months			
MOXFQ	47.6 (24.8)	48.1 (22.3)	47.9 (23.3) *n* = 37
OMAS	56.1 (29.0)	52.5 (23.3)	54.4 (26.1) *n* = 37
EQ5D5L	0.76 (0.17)	0.78 (0.17)	0.77 (0.17) *n* = 37
6 months			
MOXFQ	40.6 (27.9)	26.3 (21.0)	32.6 (25.0) *n* = 41
OMAS	67.1 (29.1)	74.0 (23.7)	70.9 (26.1) *n* = 41
EQ5D5L	0.82 (0.19)	0.90 (0.10)	0.86 (0.15) *n* = 41

Objectives 2, 3 and 4 were to evaluate the number of eligible patients within the recruiting site; evaluate the willingness of clinicians to recruit participants (the proportion of eligible patients who are offered participation in the study); evaluate the willingness of patients to be randomised (the proportion of eligible patients who agree to participate in the study). Between 10 November 2015 to 19 September 2016, 197 potential participants were screened at the recruiting site. A further eight patients attended the hospital with an ankle fracture, but were not screened as they presented at a time when research staff were not available. Of the 197 patients screened, 112 (57%) were excluded as they did not meet the eligibility criteria, the main exclusion being ‘other lower limb injury’. Thirty five of the 197 screened (18%) were eligible but declined to participate, the main reason being a preference for the functional brace. No eligible participants presented and were declined the study by the treating clinician (Fig. 1).

The remaining 50 patients were eligible and consented (25%) to take part in the study. Each arm contained 25 participants, allocated at random. There were two participants who crossed over from their allocated group; one in the plaster cast group who crossed over to receive a fixed angle removable orthotic as their cast was too tight and one patient in the orthotic group who crossed over to the plaster cast group as they did not fit into the orthotic. During the study, there were two protocol violations, in two separate situations of participants being randomised in error.

Objective 5 was to evaluate the follow-up and response rates to questionnaires. One participant in the plaster cast group withdrew before the first follow-up for non-trial-related reasons. Overall follow-up rates were 92% (46/50) at 6 weeks, 74% (37/50) at 3 months and 82% (41/50) at 6 months. This information will be important for determining future sample size calculation for the full RCT when accounting for potential loss to follow-up in the overall sample size. At the primary endpoint of 6 months, six participants in the plaster cast group were lost to follow-up and two were lost to follow-up in the functional brace group (Fig. 1).

Objectives 6 and 7 were to refine the statistical analysis plan to provide the most efficient and sensitive analysis and discuss the feasibility trial at a national consensus meeting to inform the design of a full RCT. This feasibility study was discussed at the NIHR Trauma Trials conference and Orthopaedic Trauma Society conference (January 2017) and the British Orthopaedic Foot and Ankle Society conference (September 2017). The purpose was to feedback to clinical and patient groups the trial results and lessons learned and gain feedback and consensus on a proposed full trial.

Following consultation, several changes were made to the protocol for a full RCT. There was consensus that the OMAS should be the primary outcomes measure, rather than the MOXFQ. This was decided based on patient feedback on the poor usability of the MOXFQ in comparison with the OMAS and clinical consensus that the research should be in keeping with previously funded NIHR programmes of research [19]. There was further discussion regarding the follow-up time points and these were changed to 6 weeks, 10 weeks, 16 weeks, 24 weeks, 12 months, 18 months and 24 months, with the primary time point being 16 weeks. The decision to lengthen the follow-up period was to enable us to capture the longer-term complications which may arise in this population, such as removal of metalwork. The more frequent follow-up points in the first 24 weeks post randomisation were decided to be more relevant to a trial of rehabilitation and reflected what is known about recovery time scales following this injury. The decision was also made to exclude the use of participant diaries, as this proved to have a low response rate, with the quality and usefulness of the data collected being inadequate for statistical analysis. Finally, pre-planned subgroup analysis focussing on age and op/non-operative management groups was seen as appropriate.

## Discussion

This feasibility study has provided valuable insight, enabling a greater understanding of a how a full RCT to compare plaster cast with fixed angle removable orthotic following an ankle fracture should be designed. Whilst it is not possible to draw inferences on the clinical question of effectiveness between the two interventions, and this was not the aim of the study, we are able to demonstrate the feasibility of conducting a trial that will be powered to do so.

The first objective was to evaluate the distributional properties of the MOXFQ in order to estimate the likely sample size required for a full RCT. With the data presented in the “Results” section, we have identified the standard deviation for both MOXFQ and OMAS and likely response rates. Based on this information, we calculated a conservative sample size for OMAS (OMAS chosen as the primary outcome measure following the consensus feedback presented in the Results). Based on a standard deviation of 30 points and a 10-point minimally clinically important difference, with two-sided significance set at 5% and 90% power, the required total sample size for the main study is 382 participants.

The second, third and fourth objectives related to recruitment of participants, more specifically the number of eligible patients within the recruiting site; willingness of clinicians to recruit participants and willingness of patients to be randomised. We have shown an adequate number of eligible participants at the recruiting site, demonstrating a suitable patient pool from which to recruit. We have further demonstrated clinician willingness to enter the patients into the trial, so can conclude there is sufficient clinical equipoise in this area. Most people approached were willing to participate in the study, with 59% agreeing to participate. We did notice a slightly higher proportion of patients declining as they preferred the removable orthotic. Overall, we observed an average recruitment rate of five patients randomised per month. Taking into account that recruitment is often lower outside of the lead site, we project that all participating sites should be able to recruit at a rate of three patients per month in the context of a national multi centre RCT.

Objective 5 was to evaluate the follow-up and response rates to questionnaires. The follow-up rates were used to calculate the required sample size for the larger trial, which will allow a 20% loss to follow-up. Based on the 382 sample size calculation above, allowing a margin of 20% loss during follow-up, this gives a final figure of 478 participants in total. Therefore, 239 participants randomised to each group will provide 90% power to detect a difference of 10 points in OMAS at the 5% level, allowing 20% loss to follow-up.

The final objectives were to present this feasibility study to gain consensus on the design of a planned future multi centre RCT and subsequently refine statistical analysis plans and trial-related procedures. The key changes following this process were a change of primary outcome measure from MOXFQ to OMAS and a change to the primary endpoint for the proposed larger trial.

## Conclusions

It is clear from this feasibility study that sufficient potential participants present with this injury, there is clinical equipoise, patient willingness and refined trial procedures are capable of achieving a margin within 20% loss during follow-up. Based on this combined information an application for further funding to support a full RCT was submitted and was successful. Ankle Injury Rehabilitation: A multi-centre RCT began opened to recruitment November 2017 across the UK (NIHR: CDF-2016-09-009; ISRCTN15537280).

## Abbreviations

ISRCTN: International Standard Randomised Controlled Trial Number
MOXFQ: Manchester-Oxford Foot and Ankle Questionnaire
NHS: National Health Service
NIHR: National Institute for Health Research
NRES: National Research Ethics System
OMAS: Olerud and Molander Ankle Score
ORIF: Open Reduction and Internal Fixation
RCT: Randomised controlled trial
REC: Research Ethics Committee
RfPB: Research for patient benefit
TMG: Trial Management Group
